# Gender Inequality is negatively associated with academic achievement for both boys and girls

**DOI:** 10.1038/s41539-024-00261-7

**Published:** 2024-07-26

**Authors:** Li Zhang, Baolige Chao, Yan Gao, Wenjing Wang, Yingzi Yuan, Chuangsheng Chen, Ziqiang Xin

**Affiliations:** 1 School of Sociology and Psychology, Central University of Finance and Economics, 100081 Beijing, China; 2 School of Statistics and Mathematics, Central University of Finance and Economics, 100081 Beijing, China; 3 School of Computer and Information Technology, Beijing Jiaotong University, 100044 Beijing, China; 4 Department of Psychology and Social Behavior, University of California, Irvine, Irvine, 92697 USA; 5 Department of Psychology, Renmin University of China, 100872 Beijing, China

**Keywords:** Human behaviour, Education

## Abstract

To examine the role of inequality in academic achievement, we analyse a cross-national dataset including data from three cycles from 2012 to 2018 from the PISA, an international assessment of 15-year-old students’ math, reading, and science performance. The Gini coefficient and gender inequality index (GII) were used as metrics for a country’s economic inequality and gender inequality, respectively. The results show that gender inequality has a negative association with academic achievement for both boys and girls. Moreover, gender inequality has a stronger association with academic achievement than does economic inequality. We also find that gender inequality in reproductive health may contribute substantially to the association between gender inequality and academic achievement. Despite substantial advances in gender equality worldwide, multisectoral and multilevel approaches from the community to the country level are needed to ensure substantial long-term reductions in economic, gender, and educational inequalities.

## Introduction

As a kind of potential human capital, academic achievement determines future employment, socioeconomic status, and financial stability and reflects an individual’s quality of life^1,2^. According to social-ecological theory^3^, individual differences in academic achievement can be partly attributed to socioeconomic contexts at the macrosystem level, which is characteristic of a given culture with particular reference to material resources, belief systems, customs, opportunity structures, and so on. However, compared with the abundant research on the relationship between family or school at the microsystem level and children’s academic achievement^4–6^, few educational studies have examined the relationship between economic context and children’s academic achievement. In these studies, economic development has been found to be positively associated with academic achievement^7^. Using data from the Programme for International Student Assessment (PISA), researchers reported that national foundational advancements, such as advancing to a higher income classification, are associated with significant increases in country-level PISA scores^7^.

Economic inequality, which refers to the concentration of wealth in fewer hands^8–10^, is associated with a range of negative social phenomena, including higher crime rates, lower social cohesion, and adverse physical and mental health outcomes^11,12^. To date, many studies have revealed a negative association between economic inequality and academic achievement^13–17^. For example, researchers analysed six cohorts of PISA scores from 2000 to 2015 and found that within-country economic inequality has a negative association with the academic achievement of students in that country^17^. A study analysing data from the 2006 PISA showed that more egalitarian countries (those with lower Gini coefficients) have greater average achievement, more very high-achieving students, and fewer very low-achieving students than less egalitarian countries^16^. One plausible explanation for the negative consequence of economic inequality comes from neo-materialist theory, which posits that the association between economic inequality and a range of social and psychological outcomes reflects adverse circumstances and insufficient resources for individuals^18^. Another plausible explanation comes from social psychology theories that propose that living in a highly unequal environment can intensify the sense of relative deprivation among low-income individuals, thereby leading to heightened anxiety and stress, which in turn may give rise to a series of negative outcomes^19^.

Gender inequality has also been found to be associated with academic achievement. Gender is not only a descriptive term of biological difference but also shapes social structures and relations in education, as does ethnicity and class^20^. Recently, gender has been shown to have unsettling features in comparative education^21^. Nevertheless, gender equality has typically been a desirable outcome in education^22^, and policy makers should consider interventions and outcomes through the lens of gender equality when developing education policies. Gender inequality refers to the unequal distribution of power, resources, and status among different gender groups, resulting in hierarchical power structures^23^. To date, this line of research has typically focused on the relationship between gender inequality and the gender gap in math achievement^24,25^. However, some findings do not support the idea that boys have an advantage over girls in mathematics^26,27^. For example, one study that analysed the 2015 Trends in International Mathematics and Science Study (TIMSS) identified some countries, mainly in the Middle East, as outliers with higher academic achievement by girls than boys^27^. One plausible explanation for this phenomenon could be that under the pressure of survival, girls tend to prefer mathematics-related careers with higher income returns, leading them to exert more effort and perform better in mathematics learning^28^. However, there is a growing consensus that gender differences in math performance are small or trivial in most countries^29,30^. These findings support the gender similarities hypothesis, which assumes that males and females are similar in most psychological variables^29^. Therefore, gender differences in academic achievement may not be as large as we think.

In line with this, two recent studies have shown that gender inequality is associated with academic achievement for both boys and girls^22,31^. Through the use of qualitative comparative analysis and correlational analyses, gender equality has been found to be the most relevant and consistent factor strongly associated with the 2015 PISA results in 49 countries for all children^22^. Another study reported that gender equality was consistently correlated with PISA scores at each time point from 2006 to 2018, and changes in gender equality were positively associated with changes in PISA scores for both boys and girls^31^.

However, these two studies have two noteworthy limitations. One limitation is that their analyses were performed at the country level, and it remains unknown whether their conclusions hold true based on analyses at the student level. Analyses at the country level do not account for the hierarchical structure of the data, and variance at the school level and at the student level may be masked. When results at the country level are used to draw inferences at the student level, it is possible for an ecological fallacy to occur^32^. For example, at the student level, a frequent finding is that self-concept is positively correlated with achievement in mathematics^33^. However, PISA has repeatedly reported that self-concept and achievement are negatively correlated at the country level^34,35^. Therefore, analyses at the country and student levels need to be combined. The other limitation of these studies is that the role of economic inequality is not explored when the role of gender inequality is examined. If gender inequality is separate from economic inequality, one cannot observe how they interconnect or overlap^36^. More importantly, given the abovementioned association between economic inequality and educational outcomes^13–17^, it is important for researchers and policy makers to compare the roles of multiple types of inequality^31^. This approach can provide more accurate knowledge about the relationship between gender inequality and academic achievement.

Accordingly, the present study aimed to examine the relationship between inequality and academic achievement by making two improvements to previous research. First, we combined analyses at the country and student levels to confirm the robust relationship between gender inequality and academic achievement. Specifically, for our analyses, we used multilevel modelling (i.e., HLM), which is deemed to be a suitable analytic approach for PISA^37^. Moreover, machine learning techniques were used to compare the roles of multiple inequalities in academic achievement. By doing so, we pursued our first objective of investigating whether gender inequality is harmful to children’s academic achievement for both boys and girls. Based on previous findings, we hypothesize that there is a negative relationship between gender inequality and academic achievement and that this relationship is the same for boys and girls^22,31^. Although it was first proposed over 200 years ago that inequality between the sexes is detrimental for all, even to the sex it favours^38^, possible straightforward explanations for why gender equality is beneficial for educational results remain to be identified^31^. Conceivably, gender inequality may manifest in pervasive social practices within families, educational systems, peer relationships, mass media, and culture at large. All these practices socialize children to accept gender stereotypes about their academic capabilities and performance according to social cognitive learning theory^39^. Negative stereotypes can negatively affect the performance of disadvantaged adolescents through negative expectations^40,41^, and positive stereotypes can negatively affect their performance by causing them to choke under pressure^42,43^. Consequently, it is expected that there is a negative association between gender inequality and academic achievement for both boys and girls.

The second contribution of our study is that we account for economic inequality when examining the association between gender inequality and academic achievement. Accordingly, our second objective is to compare the associations between gender inequality and economic inequality with academic achievement. We hypothesize that academic achievement has a stronger association with gender inequality than with economic inequality. Like economic inequality, gender inequality is essentially an inequality of opportunity that is derived from structural circumstances beyond the scope of individual control and responsibility^44^. Thus, gender inequality and economic inequality are often found to be closely related^45^. In areas with higher economic inequality, women tend to have lower pay, social status and labour force participation than men^45–47^. However, gender inequality and economic inequality differ in several respects. Despite limited research on their differences, we present exploratory speculation based on social capital theory^48^, which is derived from the French sociologist Pierre Bourdieu^49^. Specifically, from the perspective of individual development, economic inequality can be conceived as physical capital that can transform raw material into something profitable^50^. However, since gender is a social and cultural construct enacted through social cognitive processes that are embedded within social interactions and societal institutions^51^, gender inequality can be conceived as social capital that involves institutions, relationships, attitudes, and values that govern interactions among people^48^. Physical capital is embodied in observable materials and is wholly tangible, whereas social capital exists in the relations between persons and is thus less tangible^50^. More importantly, unlike physical capital, social capital accumulates through a myriad of repeated and varied interactions, both formal and informal; thus, it is conserved and increased through use^52^. In this sense, social capital may be more deeply rooted and may be a profound factor affecting academic achievement^53^. In a recent study using panel data from 103 countries from 2006 to 2013, a negative effect of gender equality on income inequality was found, implying the profound role of gender inequality^47^. Thus, it is expected that gender inequality, as social capital, has a greater association with academic achievement than does physical capital.

To this end, PISA scores and the Gender Inequality Index (GII) were used in this study to index academic achievement and gender inequality, respectively. The PISA is a triannual international assessment of the reading, mathematics, and science literacy and skills of 15-year-old students from numerous countries worldwide that was launched by the OECD in 1997^54^. Through the PISA, the OECD aims to provide valid, comparable, cross-national evidence of education outcomes to inform educational policy decisions^54^. However, some researchers claim that there are major technical flaws regarding the representativeness of the tests and students^55^. Critics argue that the PISA harms children and classrooms through its continuous cycle of testing and endangers the well-being of students and teachers through scripted lessons and less autonomy^56^. In pursuit of best practices in education, the PISA may cause decontextualized observations and analytical rather than empirical practice claims to be gathered^57^. There are also questions about whether the PISA is an institution-building force challenged by political and ideological neutrality^58^. Despite these concerns, the PISA has unique characteristics and advantages in terms of item construction, sampling procedures, and sample size^37^. Given that the GII was first published in 2010, we chose PISA data from 2010 onwards. Furthermore, we used PISA data from multiple years, namely, the 2012 PISA, the 2015 PISA, and the 2018 PISA data, to obtain reliable results.

The GII has been increasingly used in recent studies^59,60^. It is a composite index based on three dimensions: female reproductive health, gender empowerment and gender labour market status^61^. Since the GII has three dimensions, this study is well positioned to provide evidence regarding the domains of gender inequality with the strongest links with academic achievement. The GII is unique in that it focuses on critical issues of educational attainment, economic and political participation, and female-specific health –issues, specifically regarding female reproductive health^61^. Female reproductive health includes two indicators, maternal mortality ratio and adolescent birth ratio, which both have vital implications for women, girls, and their children. One previous study showed that these indicators were strongly associated with children’s PISA scores in 2018^31^. Moreover, the United Nations Development Programme has reported that female reproductive health is the largest contributor to gender inequality worldwide^61^. Therefore, it is expected that female reproductive health has the strongest association with academic achievement.

## **Results 1:** country-level analysis

**Fig. 1:**
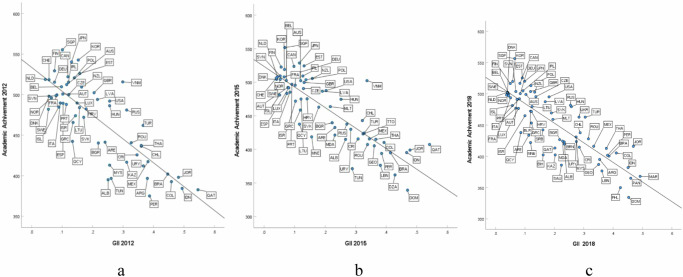
Scatter plots of academic achievement and gender inequality index. Figures (**a**), (**b**), and (**c**) show scatter plots of academic achievement versus gender inequality index for PISA2012, PISA2015, and PISA2018, respectively. The vertical axis displays the mean scores in three tested subjects (math, reading, and science) for each country in that year, while the horizontal axis represents the corresponding values of the gender inequality index.

The descriptive statistical results for the key variables are presented in Table 1. All the statistical tests reported are two-sided. First, unconditional Pearson correlation analyses at the country level were conducted to explore whether the inequality captured by the current year Gini coefficient and the GII is associated with academic achievement across the three assessment cycles from 2012 to 2015 to 2018. Figure 1 presents the correlation between academic achievement and gender inequality. Unconditional Pearson correlation analyses were also conducted separately for boys and girls. The results show that both country variables are significantly correlated with academic achievement from 2012 to 2018, regardless of gender and subject (see Table 2). Furthermore, when Fisher’s Z test was used to compare correlation coefficients, we found that the correlations between GII and academic achievement were significantly greater than those between the Gini coefficient and academic achievement (overall mean scores of the three tested subjects) in the 2012 PISA (*Fisher’s Z* = 1.95*, P* = 0.025), the 2015 PISA (*Fisher’s Z* = 3.53*, P* < 0.001), and the 2018 PISA (*Fisher’s Z* = 2.25*, P* = 0.012). These results indicate that the GII may have a stronger association with academic achievement than the Gini coefficient from 2012 to 2018. Second, stepwise regression analyses (see Table 3) of economic inequality and gender inequality for academic achievement were established at the country level. In Model 1, the Gini coefficient is included with GDP per capita as a covariate. In Model 2, the GII is added to the regression. The results of Model 1 show that economic inequality is strongly associated with academic achievement in three years: 2012, *β* = −0.49, *p* < 0.001; 2015, *β* = −0.44, *p* < 0.001; and 2018, *β* = −0.45, *p* < 0.001. However, the results of Model 2 show that once the GII is included as an independent variable, the explanatory power of the Gini coefficient disappears, and the association between economic inequality and academic achievement is no longer statistically significant: 2012, *β* = −0.02, *p* = 0.915; 2015, *β* = −0.03, *p* = 0.755; and 2018, *β* = −0.09, *p* = 0.369. In Model 2, the GII became the only significant predictor for the 2012 PISA, *β* = −0.77, *p* < 0.001; the 2015 PISA, *β* = −0.79, *p* < 0.001; and the 2018 PISA, *β* = −0.69, *p* < 0.001. Overall, 18%, 20%, and 15% of the total variance in academic achievement in 2012, 2015, and 2018, respectively, is explained by the GII.

**Table 1:** Descriptive statistics of Gender Inequality Index, Gini Coefficient, academic achievement

					Academic achievement					
	GII		Gini							
					Overall		Boys		Girls	
	*N*	*M (SD)*	*N*	*M (SD)*	*N*	*M (SD)*	*N*	*M (SD)*	*N*	*M (SD)*
2012	58	0.21(0.13)	51	35.05(6.78)	61	470.41(47.90)	61	464.66(49.49)	61	476.12(47.19)
2015	66	0.21(0.13)	55	34.47(6.52)	65	459.87(50.05)	65	454.73(52.76)	65	463.70(49.66)
2018	73	0.20(0.13)	60	34.56(6.87)	73	452.32(50.28)	73	458.68(49.76)	73	447.15(51.21)

Note: In this study, only national data from PISA were included, and regional data were excluded. Information about the participating countries can be found in Appendix Table S1. The table displays average scores in the three subjects tested, reflecting the overall academic achievement, as well as the performance of boys and girls in each country.

**Table 2:** Unconditional Pearson correlations between key variables and Academic Achievement

Variables		PISA2012		PISA2015		PISA2018	
		Gini (*N* = 51)	GII (*N* = 58)	Gini (*N* = 52)	GII (*N* = 63)	Gini (*N* = 59)	GII (*N* = 72)
Math	Overall	−0.63	−0.82	−0.53	−0.81	−0.66	−0.84
	Boys	−0.61	−0.82	−0.56	−0.86	−0.63	−0.83
	Girls	−0.65	−0.79	−0.61	−0.85	−0.68	−0.85
Reading	Overall	−0.55	−0.79	−0.43	−0.83	−0.52	−0.79
	Boys	−0.53	−0.77	−0.35	−0.75	−0.47	−0.76
	Girls	−0.58	−0.79	−0.46	−0.78	−0.57	−0.82
Science	Overall	−0.62	−0.79	−0.60	−0.85	−0.58	−0.80
	Boys	−0.61	−0.79	−0.49	−0.75	−0.54	−0.79
	Girls	−0.64	−0.77	−0.56	−0.75	−0.61	−0.77
mean	Overall	−0.63	−0.81	−0.52	−0.85	−0.60	−0.80
scores	Boys	−0.61	−0.81	−0.47	−0.84	−0.56	−0.80
	Girls	−0.65	−0.81	−0.55	−0.83	−0.63	−0.84

Note: The *p* values for the correlation coefficients are all *p* < 0.001.

**Table 3:** Stepwise regression analysis of Gender Inequality and Economic Inequality for Academic Achievement (overall)

*For the 2012 PISA (N = 49)*					
Model 1	*B*	*SE*	*β*	*t*	*P*
GDP per capita	<0.001	<0.001	0.31	2.53	0.015
Gini coefficient	−315.37	77.28	−0.49	−4.08	<0.001
*R*^2^	0.46				
Δ*R*^2^	0.44				
Model 2					
GDP per capita	<0.001	<0.001	0.02	0.13	0.900
Gini coefficient	−9.92	94.35	−0.02	−0.11	0.915
GII	−273.09	58.83	−0.77	−4.64	<0.001
*R*^2^	0.64				
Δ*R*^2^	0.61				
*For the 2015 PISA (N* *=* *50)*					
Model 1	*B*	*SE*	*β*	*t*	*P*
GDP per capita	<0.001	<0.001	0.47	4.54	<0.001
Gini coefficient	−307.33	72.27	−0.44	−4.25	<0.001
*R*^2^	0.58				
Δ*R*^2^	0.56				
model2					
GDP per capita	<0.001	<0.001	0.10	1.09	0.281
Gini coefficient	−21.73	69.17	−0.03	−0.32	0.755
GII	−284.94	44.32	−0.79	−6.43	<0.001
*R*^2^	0.78				
Δ*R*^2^	0.77				
*For the 2018 PISA (N* *=* *59)*					
model1	*B*	*SE*	*β*	*t*	*P*
GDP per capita	<0.001	<0.001	0.48	5.15	<0.001
Gini coefficient	−303.16	62.40	−0.45	−4.86	<0.001
*R*^2^	0.56				
Δ*R*^2^	0.55				
model2					
GDP per capita	<0.001	<0.001	0.14	1.39	0.169
Gini coefficient	−58.80	68.68	−0.09	−0.86	0.396
GII	−251.57	47.21	−0.69	−5.32	<0.001
*R*^2^	0.71				
Δ*R*^2^	0.70				

Note: Model 1 includes predictors for GDP per capita and the Gini coefficient, while Model 2 includes predictors for GDP per capita, the Gini coefficient, and the gender inequality index.

To test the robustness of these results, we conducted three types of robustness tests. First, we conducted stepwise regressions of the academic achievement of boys and girls at the country level. The results showed that the only significant predictor variable of academic achievement for both boys and girls was GII (Tables 4, 5). For example, in the 2018 Model 2 of the 2018 PISA, the results for boys indicate that the prediction of the Gini coefficient is not statistically significant (*β* = −0.04, *p* = 0.712), while the prediction of the GII is significant (*β* = −0.69, *p* < 0.001). For girls in the 2018 PISA study, the prediction of the Gini coefficient was not significant (*β* = −0.13, *p* = 0.183), while the prediction of the GII was significant (*β* = −0.69, *p* < 0.001). These findings show that the academic achievement of both boys and girls is negatively associated with gender inequality, and the negative association seems to be stronger than that between academic achievement and economic inequality.

**Table 4:** Stepwise regression analysis of Gender Inequality and Economic Inequality for Academic Achievement (boys)

*For the 2012 PISA (N = 49)*					
Model 1	*B*	*SE*	*β*	*t*	*P*
GDP per capita	<0.001	<0.001	0.34	2.77	0.008
Gini coefficient	−290.41	78.31	−0.45	−3.71	<0.001
*R*^2^	0.45				
Δ*R*^2^	0.42				
Model 2					
GDP per capita	<0.001	<0.001	0.05	0.44	0.662
Gini coefficient	−9.94	94.49	−0.02	−0.11	0.917
GII	−268.54	60.45	−0.77	−4.44	<0.001
*R*^2^	0.62				
Δ*R*^2^	0.60				
*For the 2015 PISA (N* *=* *50)*					
Model 1	*B*	*SE*	*β*	*t*	*P*
GDP per capita	<0.001	<0.001	0.48	4.37	<0.001
Gini coefficient	−274.77	77.63	−0.39	−3.54	<0.001
*R*^2^	0.53				
Δ*R*^2^	0.51				
Model 2					
GDP per capita	<0.001	<0.001	0.12	1.14	0.259
Gini coefficient	−6.11	79.51	0.01	0.08	0.939
GII	−280.22	50.95	−0.77	−5.50	<0.001
*R*^2^	0.72				
Δ*R*^2^	0.70				
*For the 2018 PISA (N* *=* *59)*					
Model 1	*B*	*SE*	*β*	*t*	*P*
GDP per capita	<0.001	<0.001	0.48	4.95	<0.001
Gini coefficient	−276.95	65.91	−0.41	−4.20	<0.001
*R*^2^	0.52				
Δ*R*^2^	0.50				
Model 2					
GDP per capita	<0.001	<0.001	0.14	1.29	0.204
Gini coefficient	−27.38	73.75	−0.04	−0.37	0.712
GII	−256.93	50.69	−0.69	−5.07	<0.001
*R*^2^	0.67				
Δ*R*^2^	0.65				

Note: Model 1 includes predictors for GDP per capita and the Gini coefficient, while Model 2 includes predictors for GDP per capita, the Gini coefficient, and the gender inequality index.

**Table 5:** Stepwise regression analysis of Gender Inequality and Economic Inequality for Academic Achievement (girls)

*For the 2012 PISA (N* *= 49)*					
Model 1	*B*	*SE*	*β*	*t*	*P*
GDP per capita	<0.001	<0.001	0.26	2.22	0.031
Gini coefficient	−347.78	77.66	−0.53	−4.48	<0.001
*R*^2^	0.47				
Δ*R*^**2**^	0.45				
Model 2					
GDP per capita	<0.001	<0.001	0.02	0.24	0.815
Gini coefficient	−34.93	91.56	−0.05	−0.38	0.705
GII	−279.71	58.57	−0.78	−4.78	<0.001
*R*^2^	0.65				
Δ*R*^2^	0.63				
*For the 2015 PISA (N* *=* *50)*					
Model 1	*B*	*SE*	*β*	*t*	*P*
GDP per capita	<0.001	<0.001	0.41	3.95	<0.001
Gini coefficient	−338.34	70.87	−0.49	−4.77	<0.001
*R*^2^	0.58				
Δ*R*^2^	0.56				
model2					
GDP per capita	<0.001	<0.001	0.10	1.09	0.281
Gini coefficient	−76.52	71.57	−0.11	−1.07	0.291
GII	−261.21	45.86	−0.74	−5.70	<0.001
*R*^2^	0.75				
Δ*R*^2^	0.74				
*For the 2018 PISA (N* *=* *59)*					
Model 1	*B*	*SE*	*β*	*t*	*P*
GDP per capita	<0.001	<0.001	0.47	5.23	<0.001
Gini coefficient	−330.88	60.07	−0.49	−5.51	<0.001
*R*^2^	0.59				
Δ*R*^2^	0.58				
Model 2					
GDP per capita	<0.001	<0.001	0.13	1.37	0.177
Gini coefficient	−87.66	64.93	−0.13	−1.35	0.183
GII	−250.40	44.63	−0.69	−5.61	<0.001
*R*^2^	0.74				
Δ*R*^2^	0.73				

Note: Model 1 includes predictors for GDP per capita and the Gini coefficient, while Model 2 includes predictors for GDP per capita, the Gini coefficient, and the gender inequality index.

**Fig. 2:**
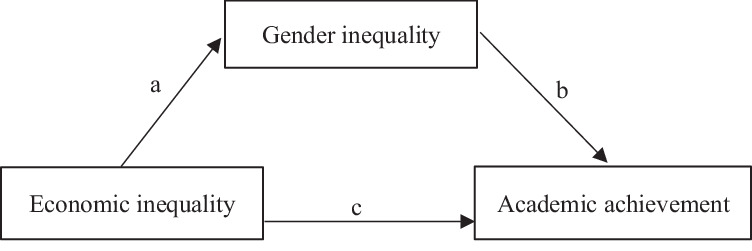
Mediation pathway graph. This graph illustrates the pathway from economic inequality to gender inequality to academic achievement. The pathway was initially identified in the stepwise regressions and later confirmed through mediation analysis.

Second, the mediation analyses (see Fig. 2 for the mediation path) show that the GII mediates the relationship between the Gini coefficient and academic achievement (see Table 6). The ratio of the indirect effect to the total effect of the Gini coefficient on academic achievement is 96.85% for the 2012 PISA, 92.93% for the 2015 PISA, and 70.22% for the 2018 PISA. These findings confirm that there is a stronger association between academic achievement and gender inequality than between academic achievement and economic inequality.

**Table 6:** Results of the mediating effect analyses: gender inequality explains the effect of economic inequality on Academic Achievement (overall)

*For the PISA2015(N* *=* *49)*							
Analysis path	*B*	*SE*	*β*	*t*	*p*	*LLCI*	*ULCI*
c	−315.37	77.28	−0.49	−4.08	<0.001	−470.91	−159.82
a	1.12	0.16	0.61	6.95	<0.001	0.01	0.02
b	−273.09	58.83	−0.78	−4.64	<0.001	−391.58	−154.60
Indirect effect	Effect	BootSE	BootLLCI	BootULCI	Indirect/total effect		
GII	−3.05	0.71	−4.38	−1.58	96.85%		
*For the PISA2015(N* *=* *50)*							
c	−307.33	72.27	−0.44	−4.25	<0.001	−452.73	−161.94
a	1.00	0.17	0.52	5.74	<0.001	0.65	1.34
b	−284.94	44.32	−0.78	−6.43	<0.001	−374.14	−195.73
Indirect effect	Effect	BootSE	BootLLCI	BootULCI	Indirect/total effect		
GII	−2.86	0.73	−4.41	−1.52	92.93%		
*For the PISA2018(N* *=* *59)*							
c	−279.99	65.85	−0.47	−4.25	<0.001	−492.74	−147.51
a	0.90	0.17	0.52	5.39	<0.001	0.56	1.23
b	−222.46	47.99	−0.65	−4.64	<0.001	−319.06	−125.86
Indirect effect	Effect	BootSE	BootLLCI	BootULCI	Indirect/total effect		
GII	−1.66	0.62	−3.27	−0.86	70.22%		

Note: This table displays the average scores of countries in the three subjects tested for the years PISA 2012, PISA 2015, and PISA 2018 as outcome variables. Gender inequality is employed as the mediating variable, while economic inequality is included as the predictor variable in the mediation analysis. Notably, upon examining the p-values, it becomes evident that all paths (a, b, and c) are statistically significant.

Finally, we investigate the causality between gender inequality and academic achievement using a fixed effects model based on country-year panel data (see Table 7). In the panel regression, we use mean scores for three tested subjects (math, reading and science) and respective scores for math, reading and science literacy to represent country-level academic achievement. The GII and Gini coefficient are used as independent variables. The GDP per capita, the country fixed effect and the year fixed effect are controlled for in the regression. The results show that the coefficients of GII in all four regression models are negatively significant, which suggests that an increase in gender inequality will significantly weaken students’ academic achievement. Specifically, for every unit increase in the GII, reading scores decrease by an average of 0.47 units, math scores decrease by an average of 0.18, and science scores decrease by an average of 0.29 units. These results suggest that gender inequality has the smallest impact on math achievement. However, as shown in each model, the Gini coefficient has no significant effect on students’ academic achievement, suggesting that economic inequality cannot explain changes in academic achievement.

**Table 7:** Fixed effect panel regression of GII and academic achievement (Overall)

	Mean scores			Math			Reading			Science		
	*β*	*SE*	*P*	*β*	*SE*	*P*	*β*	*SE*	*P*	*β*	*SE*	*P*
GII	−0.31	0.16	0.050	−0.18	0.11	0.085	−0.47	0.28	0.099	−0.29	0.14	0.039
Gini coefficient	−0.25	0.25	0.322	−0.17	0.21	0.428	−0.26	0.29	0.365	−0.32	0.30	0.281
GDP Per Capita	0.00	<0.001	0.22	0.00	<0.001	0.14	0.00	<0.001	0.452	0.00	<0.001	0.294
Constant	6.30	0.09	<0.001	6.24	0.08	<0.001	6.33	0.11	<0.001	6.33	0.11	<0.001
Country fixed effect	Yes			Yes			Yes			Yes		
Year fixed effect	Yes			Yes			Yes			Yes		
*N*	149			149			149			149		
*R*^2^	0.10			0.06			0.13			0.12		

Note: Mean scores represent the mean scores in three tested subjects.

## **Result 2:** individual-level analysis

Multilevel mixed-effects models at the student level were constructed and supported the significant association of gender inequality with academic achievement over the three cycles (Table 8, Model A). Following the PISA guidelines, sampling weights were applied to our analyses. In the model, the student level is nested at the school level, and the school level is nested at the country level. We include two student variables (gender and socioeconomic status), three school variables (location, shortage of staff, and educational resources), and three country variables (GDP per capita, Gini coefficient, and GII). As shown in the results of Model A in Table 8, GII has a significant main effect on academic achievement in all three test subjects for the 2018 PISA, suggesting that when the GII of a country decreases by 0.1 point (the extreme values are 0 and 1), the scores of students in that country increase by 16.58 to 29.36 points. This pattern persists for the 2015 PISA and the 2012 PISA, suggesting a consistent relationship between gender inequality and academic achievement over time.

**Table 8:** Results from multilevel mixed-effects models

Variables	Math			Reading			Science		
	*B*	*SE*	*P*	*B*	*SE*	*P*	*B*	*SE*	*P*
*Model A for the 2012PISA*									
Gini coefficient	−98.89	88.93	0.273	−71.33	87.05	0.417	−43.51	90.97	0.634
GII	−224.47	68.57	0.003	−136.61	57.29	0.022	−230.17	72.60	0.003
*Model B for the 2012PISA*									
Reproductive health	−52.84	31.44	0.100	−38.79	35.71	0.284	−47.21	34.48	0.179
Empowerment	113.42	96.66	0.248	95.73	81.23	0.246	74.44	93.69	0.432
Labor market	−653.22	349.02	0.068	−283.00	327.03	0.392	−753.86	383.84	0.056
*Model A for the 2015PISA*									
Gini coefficient	−132.46	76.96	0.092	87.18	75.84	0.257	−69.93	81.69	0.397
GII	−277.06	57.59	<0.001	−231.52	48.58	<0.001	−161.73	51.01	0.003
*Model B for the 2015PISA*									
Reproductive health	−146.41	50.83	0.007	−103.89	56.00	0.070	−106.18	59.50	0.081
Empowerment	−342.31	90.27	0.001	−121.64	77.99	0.126	−17.22	93.03	<0.001
Labor market	−132.62	96.30	0.176	−1.94	62.96	0.976	−253.07	64.42	0.142
*Model A for the 2018PISA*									
Gini coefficient	−71.63	53.42	0.186	91.15	44.99	0.597	−97.04	45.99	0.282
GII	−293.67	51.44	<0.001	−232.39	64.33	0.001	−165.88	48.71	0.002
*Model B for the 2018PISA*									
Reproductive health	−144.61	70.31	0.045	−174.80	76.79	0.027	−126.29	65.71	0.060
Empowerment	−274.70	63.75	0.074	−209.95	91.35	0.026	−85.50	81.95	0.302
Labor market	61.11	99.94	0.543	−4.88	75.49	0.949	−35.89	75.70	0.637

Note: The table shows the results of multilevel linear regressions for countries in each of the three subjects as outcome variables for the years PISA2012, PISA2015, and PISA2018, with Model A showing the results for the Gini coefficient and GII as key variables and Model B showing the results for the three subdimension of GII as key variables, with the full results for Models A and B for each year available in the supplementary information.

Notably, the Gini coefficient is nonsignificant for all three years. To further confirm the close relationship between GII and academic achievement, we ran “horse races” at the student level and used three machine learning techniques (LASSO, random forest, XGBoost) for model selection to compare the explanatory power of GII, GDP, and the Gini coefficient for academic achievement for the 2018 PISA. All three machine learning results (see Table 9) show that GII exhibits near-top ranking explanatory power for academic performance among the three country-level variables, consistently surpassing the predictive strength of the Gini coefficient, which is consistent with the results from the multilevel mixed-effects model. The results hold true when the data for boys’ and girls’ students are analysed separately. In summary, the machine learning results also confirm that the gender inequality captured by the GII can predict academic achievement better than the economic inequality captured by the Gini coefficient.

**Table 9:** Model selection using three machine learning techniques for the explanatory power of country-level variables in PISA2018

1. LASSO			
math-overall	GII	Gini coefficient	GDP Per Capita
Boys	GII	GDP Per Capita	Gini coefficient
girls	GII	GDP Per Capita	Gini coefficient
reading-overall	GII	Gini coefficient	GDP Per Capita
Boys	GII	Gini coefficient	GDP Per Capita
Girls	GII	Gini coefficient	GDP Per Capita
Science-overall	GII	GDP Per Capita	Gini coefficient
Boys	GII	GDP Per Capita	Gini coefficient
Girls	GII	GDP Per Capita	Gini coefficient
2. Random Forest			
math-overall	GII	GDP Per Capita	Gini coefficient
Boys	GII	Gini coefficient	GDP Per Capita
Girls	GII	GDP Per Capita	Gini coefficient
reading-overall	GII	Gini coefficient	GDP Per Capita
Boys	GII	GDP Per Capita	GDP Per Capita
Girls	GII	Gini coefficient	Gini coefficient
Science-overall	GDP Per Capita	GII	Gini coefficient
Boys	GDP Per Capita	GII	Gini coefficient
Girls	GII	GDP Per Capita	Gini coefficient
3. Xgboost			
math-overall	GDP Per Capita	GII	Gini coefficient
Boys	GII	GDP Per Capita	Gini coefficient
Girls	GII	GDP Per Capita	Gini coefficient
reading-overall	GII	GDP Per Capita	Gini coefficient
Boys	GII	GDP Per Capita	Gini coefficient
Girls	GII	GDP Per Capita	Gini coefficient
Science-overall	GDP Per Capita	GII	Gini coefficient
Boys	GDP Per Capita	GII	Gini coefficient
Girls	GII	GDP Per Capita	Gini coefficient

Note: The more forward variables there are, the better the predictive ability for academic achievement.

Finally, multilevel mixed-effects models are also constructed for the three dimensions of the GII to determine which dimension of gender inequality is more relevant for academic achievement. As shown in the results of Model B in Table 8, one main finding is that gender inequality in female reproductive health is associated with academic achievement for the 2015 PISA and the 2018 PISA. The full models are provided in Supplementary Tables 3–8.

## Discussion

This study showed that gender inequality is negatively associated with academic achievement. Gender is essentially a system of cultural beliefs about gender that influence people’s activities^62^. Among these beliefs, some may be stereotypical. For example, it is believed that girls are not as good as boys at mathematics, which is a kind of gender stereotype about academic capabilities. According to social cognitive learning theory^39^, adolescent gender stereotypes about academic capabilities and performance are shaped by pervasive practices. As institutional expressions of societal attitudes and important places for students’ socialization, schools intentionally or unintentionally communicate inequalities to adolescents^63,64^. For example, the socioeconomic characteristics of adolescents’ peers in school are significantly related to their academic achievement in Türkiye^6^. According to 20 years of comparable data for 13 countries from the TIMSS, an increased mathematics achievement gap is observed in educational systems that tend to be decentralized or reduce investment in education^4^. This process holds true, especially in countries with more liberal egalitarian societies^65^, where gender discrimination and self-identity are driven by the gender essentialist ideology (e.g., cultural beliefs in fundamental and innate gender differences) and self-expressive value systems^28,62^. An adolescent may seek better academic achievement when he or she has high efficacy expectancies and perceives a good chance of succeeding academically^66^. However, gender stereotypes in the academic domain are very common^40,67^ and may have a negative impact on adolescents’ academic achievement via expectations of their competence and performance^41^. In this vein, a negative relationship between gender inequality and academic achievement is observed in this study.

More importantly, there is a robust association between gender inequality and academic achievement for both boys and girls. This finding reflects a loss-of-function result of gender inequality for both boys and girls, leading to the conclusion that the negative aspects of gender inequality manifest at the overall societal level. One possible explanation for this finding may be that both girls and boys are subject to gender stereotypes. It is well known that negative stereotypes can disrupt the academic achievement of disadvantaged students through negative expectations^41^. However, positive expectations can also harm the performance of advantaged students when a promotion focus is activated^68^. Individuals are sensitive to positive cues and hence show impaired performance when confronted with positive expectations due to apprehensions about meeting maximal objectives. They may “choke under pressure” in the face of a positive stereotype^42^. In this way, gender inequality is negatively associated with academic achievement for both boys and girls through gender stereotypes.

Consistent with our hypothesis, gender inequality is more strongly associated with academic achievement than economic inequality is. Furthermore, according to the results of the fixed effects panel regression, gender inequality, not economic inequality, can explain the change in academic achievement from 2012 to 2018. These findings are somewhat counterintuitive since economic factors are usually found to be the most important enabling conditions in education^31^. One possible explanation is that gender inequality has a more proximal role than economic inequality in academic achievement. Moreover, gender inequality may be an enduring social consequence of economic inequality. Economic inequality tends to breed status seeking and rank competition^69,70^. Men and woman have been found to exhibit different levels of efficiency in competitive environments^71^. In this manner, economic inequality may result in an increase in gender inequality. Gender inequality further permeates from society to school, directly impacting academic achievement. According to the cultural evolution perspective of schooling, schooling can socialize students to accept a higher-payoff cultural model by immersing children in a structure of rewards and sanctions^63^. As members of both society and school, teachers pass their gender stereotypes formed in society to students. Through such transmission, gender inequality may produce an impact on students’ academic performance. Moreover, gender inequality can permeate from society to family. It is argued that gender inequality in family hinders the progress of education^38^. Mothers’ physical and mental well-being, educational attainment, and occupational standing significantly influences the academic achievement of both boys and girls^72–75^.

Economic inequality and gender inequality are intricate social constructs with intertwined yet distinct characteristics. Economic disparities exacerbate gender inequality by amplifying existing barriers faced by women in accessing education, healthcare, and economic opportunities^76,77^. Conversely, gender inequality contributes to economic disparities by impeding women’s participation in the labour force, entrepreneurship, and decision-making roles, as highlighted^61,78^. However, economic inequality and gender inequality have distinct drivers and manifestations. Economic inequality primarily stems from disparities in wealth, income, and access to resources^79^. Economic inequality involves tangible and externally observable forms of physical resources. In contrast, as a kind of social capital, gender inequality may be more deeply rooted and broadly complicated than economic inequality is. Gender inequality consists of less tangible functional social capital, including predominant attitudes, norms of behaviour and shared values within a social unit^48^. Therefore, gender inequality is often seen as a multidimensional phenomenon that implies a gender gap in health, education, income, labour and political participation^47^.

Finally, an interesting finding is that gender inequality in reproductive health may contribute greatly to the association between overall gender inequality and academic achievement. This finding is consistent with a prior study^31^ in which a reduction in the adolescent birth rate and an increase in the percentage of parliamentary seats held by women were significantly associated with a positive change in PISA scores from 2006 to 2018. Reproductive health is specific to women and reflects health risks related to pregnancy and childbirth as well as limited educational and job opportunities^61^. By defining the “child penalty” as the percentage by which women fall behind men due to children, the long-term child penalty in earnings in Denmark was close to 20% over the period 1980–2013^80^. The mental health penalties of having a child have also been observed in a Chinese sample^81^. In this study, two indicators of female reproductive health, the maternal mortality ratio and the adolescent birth ratio, essentially reflect child penalties for the health and opportunities faced by women. Therefore, it is not surprising that they contribute substantially to the influence of gender inequality on academic achievement.

In conclusion, this study reveals that gender inequality rather than economic inequality is strongly associated with academic achievement for both boys and girls. The underlying mechanism may consist of positive or negative gender stereotypes that hinder adolescents’ academic investment and performance. Therefore, appropriate policies are needed to improve gender inequality. Despite substantial reductions in gender equality worldwide, addressing gender inequality remains a global challenge because “gender inequality is deeply imbued in the norms of institutions, their decision-making processes, forms of exercising power, their rules, unwritten cultures, and approaches to allocating resources”^20^. Multisectoral and multilevel approaches from the community to the country level are needed. Economic, gender and educational equality are intertwined^64^ and constitute a multidirectional feedback system; thus, efforts in each domain and at each level are indispensable.

This study has several limitations that should be addressed in future research. First, our conclusions are restricted to the context of the use of PISA scores as indicators of academic achievement and GII scores as an indicator of gender inequality. Second, our analyses at the country level are based on academic achievement in each country, which may mask educational inequality within countries^82^. Future studies can consider the performance gap as an indicator of academic achievement by calculating the difference between high-performing and low-performing students within each country to obtain a more comprehensive understanding of academic achievement. Third, this study investigates gender as a noun when considering gender differences in education systems. Gender can be understood as an adjective when considering how gendered relations of power, distribution of resources, and forms of struggle occur in educational settings or as a verb when considering mixed gender identities, performances or actions and the space of education as active and changing in its movement between times, spaces and discourses^21^. Finally, although we explored causality using the fixed-effect model, we should be cautious in explaining the causal associations detected in this study given the cross-sectional nature of PISA data at the student level and the nonexperimental design of the study.

## Methods

### The programme for international student assessment

PISA data are obtained from students, parents, teachers, and school principals through the completion of academic assessments and self-reported contextual questionnaires. This assessment measures the ability of 15-year-olds to use their reading, mathematics, and science knowledge and skills to meet real-life challenges^83^. It assesses whether national and regional education models or the quality of education have achieved the goal of preparing students to become well-rounded individuals for modern society^83^. Since 1997, the PISA has published a total of seven cycles of data collection at a triennial measurement frequency. Each wave of data includes students’ mathematical, reading, and science literacies, which are fixed from year to year. Other literacies and students’ information on learning backgrounds change from year to year. We used data from the 2012 PISA, the 2015 PISA, and the 2018 PISA, which include 65, 72, and 79 countries and economies, respectively, covering a total of 1,569,379 students. In this study, not all countries were covered due to the absence of key variables for some countries and economies. Detailed sample information can be found in Supplementary Table 1. The correlations between country variables can be found in Supplementary Table 2.

### PISA methodology, plausible values, and sampling weights

To mitigate the impact of student fatigue and the constraints of time available for testing and to encompass a broader spectrum of measurements, the PISA employs item response theory (IRT) models for scaling. Rather than responding to all the questions in a particular administration, students are tasked with answering a subset of questions selected from a larger pool. The probability distributions of their ability levels are then estimated based on their responses to this specific subset of questions. Therefore, the cognitive item scores provided by the PISA do not represent students’ actual scores on those items. Instead, they constitute several randomly selected values derived from an estimated distribution of students’ ability levels, referred to as ‘plausible values’. According to the 2012 PISA, five plausible value scores were assigned per cognitive item, while the 2015 and 2018 PISA offered ten scores per item. In this study, we performed analyses at both the country and individual levels. At the country level, we employed the PISA-endorsed code generator data analysis software IDB Analyzer to calculate national academic achievement scores through plausible value computations. These outcomes are also detailed in the official reports published by the PISA. For individual-level analysis, we utilized Hierarchical Linear and Nonlinear Modeling (HLM) software, which is specialized software designed to dissect complex multilevel data structures and facilitate the calculation of plausible values.

The PISA sampling process involves two steps, namely, selecting a sample of schools containing the target group for the study and then randomly selecting 35 students from the chosen schools. However, the probability of selection for each student is not uniform and varies with the school’s size. Larger schools with more students have a lower probability of student selection, and vice versa. This can result in undersampling or oversampling issues within participating schools. To rectify this, sampling weights are adjusted for each student and school in the sample. In this study, individual-level analyses were conducted, applying sampling weights at both the student and school levels to address bias.

Ethical approval and informed consent are not applicable to this study because the data used here are from publicly available databases (PISA, World Bank, and United Nations Development Program) and no experiments were conducted.

### Overview of key variables

Academic achievement. The definition of academic achievement is contingent upon the indicators employed for its measurement^84^. In this study, we analysed data based on three cycles of the PISA from 2012 to 2018 using areas of academic achievement (math, reading, science) as independent variables. In both the country-level and student-level analyses, we used the literacy scores for mathematics, reading, and science as outcome variables. In the country-level analyses, the mean scores of the three tested subjects were also analysed.

Economic Inequality. The Gini coefficient is the most common measure of economic inequality^85^. The Gini index measures the area between the Lorenz curve and a hypothetical line of absolute equality, which is expressed as a percentage of the maximum area under the line^86^. A Lorenz curve plots the cumulative percentages of total income received against the cumulative number of recipients, starting with the poorest individual. A Gini index of 0 represents perfect equality, while an index of 100 implies perfect inequality^87^. We chose the widely used and recognizable Gini coefficient calculated by the World Bank^87^, which is based on key household survey data obtained from government statistical agencies and World Bank country departments.

Gender Inequality. The data on gender inequality were derived from the GII reported in the ‘Human Development Report’ of the UNDP. This index, which was first published in 2010, is a composite indicator that quantifies the losses a country experiences as a result of gender inequality^61^ and maintains the frequency of annual measurements published in the following year’s ‘Human Development Report’. The GII consists of five indicators in three dimensions. The female reproductive health dimension is a synthesis of the maternal mortality rate and the adolescent fertility rate; the empowerment dimension is a combination of the share of seats in parliament and the population with at least some secondary education; and the labour force dimension is calculated from the labour force participation rate. Five indicators are reported in the ‘Human Development Report’^88–90^. We calculated gender inequality in the three dimensions according to the formula used to calculate the GII. The specific calculations can be found in Method 5.

### Overview of covariates

In this study, seven covariates were considered: two at the student level, four at the school level, and one at the country level. These covariates were sourced from the PISA database for both the student and school levels and from the World Bank database for the country level.

Gender: Gender was coded as 1 for girls and 2 for boys across all three cycles of the database.

The index of economic, cultural, and social status (ESCS): The ESCS serves as a measure of students’ access to family resources, including financial capital, social capital, cultural capital, and human capital. It reflects the social position of the student’s family or household and can be interpreted as an approximation of an individual’s ranking in national and global society. The ESCS is derived from the combination of parents’ educational level (in years), parents’ occupational status on the International Socio-Economic Index (ISEI) scale, and family wealth.

For the HLM analyses for each year, three school-level covariates were considered. The difference lies in the specific covariates employed in the 2012, 2015, and 2018 analyses. In the 2018 and 2015 analyses, the school-level covariates consisted of school location, shortage of materials, and shortage of educational staff. In contrast, the 2012 analysis employed school location, shortage of teachers, and student-teacher ratio, as shortages of materials were not assessed in the 2012 PISA.

School location: The school principal provided information regarding the school’s location by responding to the following question: ‘Which of the following definitions best describes the community in which your school is located?’ with options ranging from 1 for ‘village’ to 5 for ‘large city’.

Shortage of materials: School principals reported on the extent to which the school’s capacity to provide instruction is hindered by the shortage or inadequacy of various resources, including science laboratory equipment, instructional materials such as textbooks, computers for instruction, internet connectivity, computer software for instruction, library materials, and audiovisual resources. These responses were aggregated to create a composite index of material resources, with an average of zero and a standard deviation of one for OECD countries. Higher values of this index indicate fewer hindrances to instruction due to resource shortages.

Shortages of educational staff: School principals’ assessments of the extent to which instruction in their school is hindered by a lack of qualified teachers and staff in key areas were used to create a composite index of teacher shortages. This index also had an average of 0 and a standard deviation of 1 for OECD countries, with higher values indicating a perception of more problems with instruction due to teacher shortages.

Student-teacher ratio: The student-teacher ratio was calculated by dividing the school size by the total number of teachers. Part-time teachers were weighted by 0.5, and full-time teachers were weighted by 1.0 in the computation of this index.

The GDP per capita, which is calculated as the gross domestic product divided by the midyear population, serves as a critical economic indicator. The GDP itself represents the sum of gross value added by all resident producers in the economy, including any product taxes and minus any subsidies not accounted for in the value of the products. This calculation does not factor in deductions for the depreciation of fabricated assets or the depletion and degradation of natural resources. The data are presented in current U.S. dollars. For more detailed information, please refer to the following link: https://data.worldbank.org/indicator/NY.GDP.PCAP.CD.

### Calculation methods for the three subdimensions of the GII

The GII comprises five indicators organized into three dimensions. The female reproductive health dimension combines the maternal mortality rate and the adolescent fertility rate, the empowerment dimension incorporates the share of seats in parliament with the share of the population with at least some secondary education, and the labour force dimension is derived from the labour force participation rate. These five indicators are reported in the ‘Human Development Report’. To calculate gender inequality across these three dimensions, we followed the formula used for computing the GII. The specific calculation method is outlined as follows.

First, we aggregate across dimensions within each gender group using geometric means. For the productive health dimension, the aggregation formula for females is *G*_*F*_ = $\sqrt{(\frac{10}{{MMR}}\times \frac{1}{{ABR}})}$; the formula for males is *G*_*M*_ = 1. For the empowerment dimension, the aggregation formula for females is *G*_*F*_ = $\sqrt{{PR}(F)\times {SE}(F)}$; the formula for males is *G*_*M=*_$\sqrt{{PR}(M)\times {SE}(M)}$. For the labour market dimension, the aggregation formula for females is G_F_ = LFPR(F); the formula for males is *G*_*M*_ = *LFPR(M)*.

Second, we aggregate across gender groups using a harmonic mean. For all dimensions, the following equation is used: *HARM* (*G*_*F*_, *G*_*M*_) = [((*G*_*F*_)^−1^ + (*G*_*M*_)^−1^)/2]^−1^.

Third, we calculate the geometric mean of the arithmetic means for each indicator. For the productive health dimension, the following equation is used: $\bar{{Productive\; healt}h}$ = ($\,\sqrt{(\frac{10}{{MMR}}\times \frac{1}{{ABR}})}$ + 1)/2. For the empowerment dimension, the following equation is used: $\overline{{Empowermen}{\rm{t}}}$ = ($\sqrt{{PR}(F)\times {SE}(F)}$ + $\sqrt{{PR}(M)\times {SE}(M)}$)/2. For the labour market dimension, the following equation is used: $\overline{{Labor\; market}}$ = (*LFPR(F)* + *LFPR(M)*)/2.

Finally, Gender inequality in productive health = 1 − *HARM* (*G*_*F*,_ *G*_*M*_)/$\overline{{Healt}h}$. Gender inequality in empowerment *=* 1 − *HARM* (*G*_*F*,_ *G*_*M*_)/$\overline{{Empowerment}}$. Gender inequality in the labour market = 1− *HARM* (*G*_*F*,_ *G*_*M*_)/$\overline{{Labor\; market}}$.

### Reporting summary

Further information on research design is available in the Nature Research Reporting Summary linked to this article.

### Supplementary information

Supplementary Information

Reporting Summary

## Data Availability

PISA data are available at https://www.oecd.org/pisa/data/. Gender Inequality Index are available in UNDP’s Human Development Report which can be found at https://hdr.undp.org/reports-and-publications. Gini coefficients are available at https://data.worldbank.org/indicator/SI.POV.GINI. GDP per capita are available at https://data.worldbank.org/indicator/NY.GDP.PCAP.CD. All codes used for the analyses are available by request directed tothe corresponding author.

## Code availability

All codes used for the analyses are available by request directed to the corresponding author.
