# Supplementary material for: Gender Inequality is negatively associated with academic achievement for both boys and girls
Source: NPJ Sci Learn. 2024 Jul 26;9:49. doi: 10.1038/s41539-024-00261-7 (PMC11282183; doi:10.1038/s41539-024-00261-7)
Supplement: Supplementary file 1 — Supplementary Information [file 41539_2024_261_MOESM1_ESM.pdf]

Supplementary Information

Supplementary Table 1-8

Supplementary Table 1 PISA 2012-2018 participating countries and the values of key variables for these countries

Country	Academic Achivement*			Gender Inequality Index			Gini Coefficient		
	2012	2015	2018	2012	2015	2018	2012	2015	2018
Albania	395	415	420	0.251	0.267	0.234	29.0	32.8	30.1
algeria	a	362	a	a	0.429	a	a	c	a
Argentina	397	b	395	0.38	c	0.354	41.3	c	41.3
**Australia**	512	502	499	0.115	0.12	0.103	c	c	34.3
**Austria**	501	492	491	0.102	0.078	0.073	30.5	30.5	30.8
Belarus	a	a	472	a	a	0.119	a	a	25.2
**Belgium**	510	503	500	0.098	0.073	0.045	27.5	27.7	27.2
Bosnia and Herzegovi	a	a	402	a	a	0.162	a	a	c
Brazil	402	395	400	0.447	0.414	0.386	53.4	51.9	53.9
Brunei Darussalam	a	a	423	a	a	0.234	a	a	a
Bulgaria	440	440	427	0.219	0.223	0.218	36.0	38.6	41.3
**Canada**	522	524	517	0.119	0.098	0.083	33.5	33.7	32.5
**Chile**	436	443	438	0.36	0.322	0.288	a	44.4	c
Colombia	393	410	405	0.459	0.393	0.411	52.6	51.0	50.4
Costa Rica	426	416	415	0.346	0.308	0.285	48.4	48.4	48.0
Croatia	482	475	472	0.179	0.141	0.122	32.5	31.1	29.7
Cyprus	442	438	438	0.134	0.116	0.086	34.3	34.0	32.7
**Czech Republic**	500	491	495	0.122	0.129	0.137	26.1	25.9	25.0
**Denmark**	498	504	501	0.057	0.041	0.04	27.8	28.2	28.2
Dominican Republic	a	339	334	a	0.47	0.453	a	45.2	43.7
**Estonia**	526	524	525	0.158	0.131	0.091	32.9	32.7	30.3
**Finland**	529	523	516	0.075	0.056	0.05	27.1	27.1	27.3
**France**	500	496	494	0.083	0.102	0.051	33.1	32.7	32.4
Georgia	a	405	387	a	0.361	0.351	a	36.5	36.4
**Germany**	515	508	500	0.075	0.066	0.084	31.1	31.4	31.8
**Greece**	466	459	453	0.136	0.119	0.122	36.3	36.0	32.9
**Hungary**	486	475	479	0.256	0.252	0.258	30.8	30.4	29.6
**Iceland**	485	481	481	0.089	0.051	0.057	26.8	26.8	c
Indonesia	384	395	382	0.494	0.467	0.451	40.5	40.4	38.4
**Ireland**	515	509	505	0.121	0.127	0.093	33.2	31.8	30.6
**Israel**	474	472	465	0.144	0.103	0.1	41.6	39.7	38.6
**Italy**	490	485	477	0.094	0.085	0.069	35.2	35.4	35.2
**Japan**	540	529	520	0.131	0.116	0.099	c	c	c
Jordan	398	399	416	0.482	0.478	0.469	c	c	c
Kazakhstan	417	b	402	0.312	0.202	0.203	28.2	26.8	27.8

**Korea**	543	519	520	0.153	0.067	0.058	31.6	c	c
Kosovo	a	362	361	a	c	c	a	26.5	c
**Latvia**	494	487	487	0.216	0.191	0.169	35.2	34.2	35.1
Lebanon	a	376	377	a	0.381	0.362	a	c	c
Liechtenstein	525	a	a	c	a	a	c	a	a
**Lithuania**	484	475	480	0.157	0.121	0.124	35.1	37.4	35.7
**Luxembourg**	490	483	477	0.149	0.075	0.078	34.3	32.9	35.4
Malaysia	413	b	431	0.256	0.291	0.274	c	41.1	41.2
Malta	a	464	459	a	0.217	0.195	a	29.4	28.7
**Mexico**	417	416	416	0.382	0.345	0.334	48.7	c	46.7
Moldova	a	421	424	a	0.232	0.228	a	27.0	25.7
Montenegro	414	419	422	c	0.156	0.119	41.2	39.0	36.8
Morocco	a	a	368	a	a	0.492	a	a	c
**Netherlands**	519	508	502	0.045	0.044	0.041	27.6	28.2	28.1
**New Zealand**	509	506	503	0.164	0.158	0.133	32.4	c	c
North Macedonia	a	369	400	a	c	0.145	a	35.6	33.0
**Norway**	496	504	497	0.065	0.053	0.044	25.7	27.5	27.6
Panama	a	a	365	a	a	0.46	a	50.8	49.2
Peru	375	394	402	0.387	0.385	0.381	44.4	43.4	42.4
Philippines	a	a	350	a	a	0.425	a	44.6	42.3
**Poland**	521	504	513	0.14	0.137	0.12	33.0	31.8	30.2
**Portugal**	488	497	492	0.114	0.091	0.081	36.0	35.5	33.5
Qatar	383	407	413	0.546	0.542	0.202	c	c	c
Romania	441	407	428	0.327	0.339	0.316	36.5	35.9	35.8
Russian Federation	481	438	482	0.312	0.271	0.255	40.7	37.7	37.5
Saudi Arabia	a	a	386	a	a	0.224	a	a	c
Serbia	447	a	442	c	a	0.161	39.9	40.5	35.0
Singapore	555	552	556	0.101	0.068	0.065	c	c	c
**Slovak Republic**	472	463	469	0.171	0.179	0.19	26.1	26.5	25.0
**Slovenia**	499	509	504	0.08	0.053	0.069	25.6	25.4	24.6
**Spain**	489	492	480	0.103	0.081	0.074	35.4	36.2	34.7
**Sweden**	482	496	502	0.055	0.048	0.04	27.6	29.2	30.0
**Switzerland**	518	506	498	0.057	0.04	0.037	31.6	32.3	33.1
Thailand	437	415	413	0.360	0.366	0.377	39.3	36.0	36.4
Trinidad Tobago	a	423	a	a	0.324	a	a	c	a
Tunisia	397	371	a	0.261	0.289	a	c	32.8	a
**Turkey**	462	424	463	0.366	0.328	0.305	40.2	42.9	41.9
Ukraine	a	a	463	a	a	0.284	a	a	26.1
United Arab Emirates	441	433	434	0.241	0.232	0.113	c	c	26.0
**United Kingdom**	502	500	504	0.205	0.131	0.119	33.1	33.3	33.7
**United States**	492	488	495	0.256	0.203	0.182	40.9	41.2	41.4
Uruguay	412	430	424	0.367	0.284	0.275	39.9	40.1	39.7
Vietnam	516	502	b	0.299	0.337	0.314	35.6	c	35.7

The Academic Achievement* presented in this table is the country’s average score on the three literacies-math, reading, and science-

for that year. a Represents a country that did not participate in PISA that year. b Represents the country's technical problems with its data prevent results from being reported. c Represents a country for which the GII or Gini coefficient was not reported. OECD countries are shown in bold.

Supplementary Table 2 Correlation between GII, Gini Coefficient, and GDP per Capita in PISA Participating Countries (2012-2018)

	2012		2015		2018	
	GII	N	GII	N	GII	N
Gini coefficient	0.77	49	0.70	52	0.68	60
GDP per capita	-0.5	58	-0.59	66	-0.66	73

The p values for the correlation coefficients are all $p<0.01$.

Supplementary Table 3 The multilevel mixed-effects model for the PISA 2012

**Variables**	**Math**			**Reading**			**Science**		
	B	*SE*	*P*	*B*	*SE*	*P*	*B*	*SE*	*P*
Student-level variable									
N=321431									
Gender	11.58	2.57	<0.001	30.00	1.45	<0.001	1.95	1.58	0.219
Socioeconomic status	12.53	2.42	<0.001	11.53	2.53	<0.001	12.32	2.53	<0.001
School-level variables									
N=12725									
Location	6.58	2.11	0.002	9.84	1.87	<0.001	5.36	2.18	0.014
Shortage of staff	-0.14	0.12	0.220	-0.21	0.17	0.242	-0.24	0.13	0.055
Ratio of students to teachers	-0.15	0.12	0.220	-8.41	1.83	<0.001	-7.73	1.68	<0.001
Country-level variables									
N=46									
GDP per capita	<0.001	<0.001	0.738	<0.001	<0.001	0.368	<0.001	<0.001	0.421
**Gini coefficient**	-98.89	88.93	0.273	-71.33	87.05	0.417	-43.51	90.97	0.634
**GII**	-224.47	68.57	0.003	-136.61	57.29	0.022	-230.17	72.60	0.003
Intercept	468.22	7.32	<0.001	469.61	6.38	<0.001	475.93	7.35	<0.001

From this table, it can be found that the GII is the only country-level variable that predicts academic achievement with P < 0.001 in all test subjects.

Supplementary Table 4 The multilevel mixed-effects model for the PISA 2015

Variables	Math			Reading			Science		
	B	*SE*	*P*	*B*	*SE*	*P*	*B*	*SE*	*P*
Student-level variable									
N= 301667									
Gender	10.76	2.39	<0.001	-21.19	0.29	<0.001	5.40	1.53	0.001
Socioeconomic status	12.11	1.65	<0.001	16.91	0.16	<0.001	11.11	2.23	<0.001
School-level variables									
N= 11005									
Location	<0.001	2.31	0.013	<0.001	<0.001	0.812	<0.001	<0.001	0.043
Shortage of staff	-5.99	1.10	<0.001	-4.42	0.54	<0.001	-6.55	1.15	<0.001
Shortages of educational resources	-6.27	1.26	<0.001	-4.32	0.52	<0.001	-5.96	1.27	<0.001
Country-level variables									
N=48									
GDP per capita	<0.001	<0.001	0.474	<0.001	<0.001	0.456	<0.001	<0.001	0.733
**Gini coefficient**	-132.46	76.96	0.092	87.18	75.84	0.257	-69.93	81.69	0.397
**GII**	-277.06	57.59	<0.001	-231.52	48.58	<0.001	-161.73	51.01	0.003
Intercept	458.25	4.98	<0.001	464.02	3.41	<0.001	462.15	4.68	<0.001

It can be found that the GII is the only country-level variable that predicts academic achievement with $P < 0.05$ in all test subjects.

Supplementary Table 5 The multilevel mixed-effects model for the PISA 2018

Variables	Math			Reading			Science		
	B	*SE*	*P*	*B*	*SE*	*P*	*B*	*SE*	*P*
Student-level variable									
N= 428538									
Gender	5.16	2.95	0.072	-18.72	1.75	<0.001	2.71	0.98	0.006
Socioeconomic status	11.46	3.35	0.001	9.202	2.70	0.001	11.93	2.77	<0.001
School-level variables									
N=16521									
Location	2.62	1.42	0.067	<0.001	2.08	<0.001	<0.001	<0.001	<0.001
Shortage of staff	-3.07	1.95	0.003	-0.89	1.41	0.527	-8.75	1.43	<0.001
Shortages of educational Resources	-9.81	1.61	<0.001	-10.96	1.88	<0.001	-7.65	1.41	<0.001
Country-level variables									
N=58									
GDP per capita	<0.001	<0.001	0.416	<0.001	<0.001	0.941	<0.001	<0.001	0.359
**Gini coefficient**	-71.63	53.42	0.186	91.15	44.99	0.597	-97.04	45.99	0.282
**GII**	-93.67	51.44	<0.001	-232.39	64.33	0.001	-165.88	48.71	0.002
Intercept	461.96	5.45	<0.001	451.40	5.32	<0.001	455.16	4.60	<0.001

It can be seen that students' performance in math is negatively predicted by both the Gini coefficient and GII, while performance in the other two test subjects is only predicted by the GII.

Supplementary Table 6 The model with GII subdimensions as the main variables for PISA 2012

**Variables**		**Math**			**Reading**			**Science**	
	B	*SE*	*P*	*B*	*SE*	*P*	*B*	*SE*	*P*
Student-level variable									
Gender	1.94	1.58	0.219	-30.00	1.45	<0.001	1.94	1.58	0.219
ESCS	12.32	2.53	<0.001	11.52	2.53	<0.001	12.32	2.53	<0.001
School-level variables									
Location	6.59	2.10	0.002	9.85	1.87	<0.001	5.37	2.18	0.014
Shortage of staff	-0.15	0.12	0.219	-8.41	1.82	<0.001	-0.25	0.13	0.054
Ratio of students	-7.38	1.52	<0.001	-0.20	0.17	0.242	-7.73	1.67	<0.00
to teachers									1
Country-level variables									
GDP per capita	<0.001	<0.001	0.530	<0.001	<0.001	0.120	<0.001	<0.001	0.530
Gini coefficient	-237.71	113.22	0.042	-156.92	115.71	0.183	-181.22	117.27	0.042
**Reproductive health**	-52.84	31.44	0.100	-38.79	35.71	0.284	-47.21	34.48	0.179
**Empowerment**	113.42	96.66	0.248	95.73	81.23	0.246	74.44	93.69	0.432
**Labor market**	-653.22	349.02	0.068	-283.00	327.03	0.392	-753.86	383.84	0.056
Intercept	463.43	5.57	<0.001	466.62	5.58	<0.001	471.35	5.99	<0.001

The sample sizes at all levels are consistent with those in Supplementary Table 3.

Supplementary Table 7 The model with GII subdimensions as the main variables for the PISA 2015

Variables	Math			Reading			Science		
	B	*SE*	*P*	*B*	*SE*	*P*	*B*	*SE*	*P*
Student-level variable									
Gender	10.76	2.39	<0.001	-21.19	0.29	<0.001	5.40	1.53	0.001
ESCS	12.10	1.64	<0.001	16.91	0.16	<0.001	11.10	2.22	<0.001
School-level variables									
Location	<0.001	<0.001	<0.001	<0.001	<0.001	0.878	3.97	2.53	0.116
Shortage of staff	-4.88	1.23	0.515	-4.40	0.54	<0.001	-6.53	1.15	<0.001
Shortage of educational resource	-5.98	1.10	<0.001	-4.32	0.52	<0.001	-5.96	1.27	<0.001
Country-level variables									
GDP per capita	<0.001	<0.001	0.140	<0.001	<0.001	0.146	<0.001	<0.001	0.001
Gini coefficient	-123.44	79.37	0.127	9.10	92.67	0.923	-69.93	79.04	0.382
**Reproductive health**	-146.41	50.83	0.007	-103.89	56.00	0.070	-106.18	59.50	0.081
**Empowerment**	-342.31	90.27	0.001	-121.64	77.99	0.126	-17.22	93.03	<0.001
**Labor market**	-132.62	96.30	0.176	-1.94	62.96	0.976	-253.07	64.42	0.142
Intercept	454.04	2.75	<0.001	463.84	3.88	<0.001	458.77	2.90	<0.001

The samples at all levels are consistent with those in Supplementary Table 4.

Supplementary Table 8 The model with GII subdimensions as the main variables for the PISA 2018

**Variables**	**Math**			**Reading**			**Science**		
	B	*SE*	*P*	*B*	*SE*	*P*	*B*	*SE*	*P*
Student-level variable									
Gender	5.63	3.24	0.082	-18.72	1.75	<0.001	2.71	0.98	0.006
ESCS	11.47	3.35	0.001	9.20	2.70	0.001	11.93	2.78	<0.001
School-level variables									
Location	2.616	2.31	0.067	<0.001	<0.001	0.002	<0.001	<0.001	0.001
Shortage of staff	-3.07	1.95	0.115	-0.89	1.41	0.530	-0.84	1.29	0.515
Shortage of educational resource	-9.83	1.60	<0.001	-10.98	1.88	<0.001	-8.76	1.43	<0.001
Country-level variables									
GDP per capita	<0.001	<0.001	0.454	<0.001	<0.001	0.374	<0.001	<0.001	0.17
Gini coefficient	-135.20	58.86	0.026	81.40	69.32	0.246	-64.97	55.80	0.248
**Reproductive health**	-144.61	70.31	0.045	-174.80	76.79	0.027	-126.29	65.71	0.060
**Empowerment**	-274.70	63.75	0.074	-209.95	91.35	0.026	-85.50	81.95	0.302
**Labor market**	61.11	99.94	0.543	-4.88	75.49	0.949	-35.89	75.70	0.637
Intercept	451.63	6.39	<0.001	447.57	4.55	<0.001	451.63	6.39	<0.001

The samples at all levels are consistent with those in Supplementary Table 5.
