# Supplementary material for: Gender Inequality is negatively associated with academic achievement for both boys and girls
Source: NPJ Sci Learn. 2024 Jul 26;9:49. doi: 10.1038/s41539-024-00261-7 (PMC11282183; doi:10.1038/s41539-024-00261-7)
Supplement: Supplementary file 2 — Reporting Summary [file 41539_2024_261_MOESM2_ESM.pdf]

nature portfolio

Corresponding author(s): DAPR 00817

Last updated by author(s): June-1-2024

Reporting Summary

Nature Portfolio wishes to improve the reproducibility of the work that we publish. This form provides structure for consistency and transparency in reporting. For further information on Nature Portfolio policies, see our Editorial Policies and the Editorial Policy Checklist.

Statistics

For all statistical analyses, confirm that the following items are present in the figure legend, table legend, main text, or Methods section.

n/a	Confirmed	
☐	☒	The exact sample size (n) for each experimental group/condition, given as a discrete number and unit of measurement
☐	☒	A statement on whether measurements were taken from distinct samples or whether the same sample was measured repeatedly
☐	☒	The statistical test(s) used AND whether they are one- or two-sided<br>*Only common tests should be described solely by name; describe more complex techniques in the Methods section.*
☐	☒	A description of all covariates tested
☐	☒	A description of any assumptions or corrections, such as tests of normality and adjustment for multiple comparisons
☐	☒	A full description of the statistical parameters including central tendency (e.g. means) or other basic estimates (e.g. regression coefficient) AND variation (e.g. standard deviation) or associated estimates of uncertainty (e.g. confidence intervals)
☐	☒	For null hypothesis testing, the test statistic (e.g. F, t, r) with confidence intervals, effect sizes, degrees of freedom and P value noted<br>*Give P values as exact values whenever suitable.*
☒	☐	For Bayesian analysis, information on the choice of priors and Markov chain Monte Carlo settings
☐	☒	For hierarchical and complex designs, identification of the appropriate level for tests and full reporting of outcomes
☐	☒	Estimates of effect sizes (e.g. Cohen's d, Pearson's r), indicating how they were calculated

Our web collection on statistics for biologists contains articles on many of the points above.

Software and code

Policy information about availability of computer code

Data collection	The data used in this study are publicly available data from PISA, the World Bank and UNDP which can be found and downloaded from their official websites.
Data analysis	The data analysis software we used were SPSS, Stata, HLM6.08, IDB analyzer,vscode, python3.9.2.

For manuscripts utilizing custom algorithms or software that are central to the research but not yet described in published literature, software must be made available to editors and reviewers. We strongly encourage code deposition in a community repository (e.g. GitHub). See the Nature Portfolio guidelines for submitting code & software for further information.

Data

Policy information about availability of data

All manuscripts must include a data availability statement. This statement should provide the following information, where applicable:

- Accession codes, unique identifiers, or web links for publicly available datasets
- A description of any restrictions on data availability
- For clinical datasets or third party data, please ensure that the statement adheres to our policy

The data used in this study are publicly available data from PISA, the World Bank and UNDP which can be found and downloaded from their official websites.

Research involving human participants, their data, or biological material

Policy information about studies with human participants or human data. See also policy information about sex, gender (identity/presentation), and sexual orientation and race, ethnicity and racism.

Reporting on sex and gender	We use the terms sex and gender carefully in order to avoid confusing both terms. Our findings apply to both sexes; Sex and gender were considered in our study design; Sex was determined based on individual self-reporting. We provide disaggregated sex and gender data in the source data. We report sex-based analyses where performed. The data for this study was sourced from the PISA project administered by the OECD. Gender categorization in this dataset is binary, with girls coded as 1 and boys coded as 2, based on students' self-selection and reporting. In the PISA assessments of 2012, 2015, and 2018, a total of approximately 820,203 female students and 821,133 male students were enrolled.
Reporting on race, ethnicity, or other socially relevant groupings	PISA has released the synthesized ESCS index, which serves as a metric quantifying students' access to family resources, encompassing financial capital, social capital, cultural capital, and human capital. These elements collectively establish the social standing of a student's family or household, and can be construed as an approximation of an individual's position within both national and global society. This index is the composition of the parent's educational level (in years), the parents' occupational status on the "International Socio-Economic Index" (ISEI) scale, and family wealth.
Population characteristics	not applicable
Recruitment	not applicable
Ethics oversight	not applicable

Note that full information on the approval of the study protocol must also be provided in the manuscript.

Field-specific reporting

Please select the one below that is the best fit for your research. If you are not sure, read the appropriate sections before making your selection.

☐ Life sciences ☒ Behavioural & social sciences ☐ Ecological, evolutionary & environmental sciences

For a reference copy of the document with all sections, see nature.com/documents/nr-reporting-summary-flat.pdf

Behavioural & social sciences study design

All studies must disclose on these points even when the disclosure is negative.

Study description	Quantitative analysis of secondary data[illegible]PISA[illegible]
Research sample	We analyzed data from PISA 2012, PISA 2015, and PISA 2018. In these three years of testing, the number of participating countries and regions was 65, 73, and 79, respectively. We excluded regional data due to its limited representativeness. And in the case of certain countries, PISA has determined that they should not participate in international comparisons due to technical issues affecting their results. Consequently, the final sample size of countries participating in this study is 61, 67, and 72, respectively.
Sampling strategy	In the PISA sampling process, two main steps are involved. Firstly, a sample of schools is selected, which contains the target group for the study. Secondly, within these selected schools, 35 students are chosen randomly. However, in practice, the probability of selection isn't uniform for every student. It varies depending on the school's size; larger schools with more students have a lower likelihood of a student being selected, and vice versa. This can lead to issues of under-sampling or over-sampling in participating schools. To address this problem, sampling weights must be adjusted for each student and school within the sample. In this study, we conducted individual-level analyses using sampling weights at both the student and school levels to correct for the aforementioned bias.
Data collection	not applicable
Timing	not applicable
Data exclusions	Data of some countries have been excluded based on two reason. First, none of the PISA participating regions were included in the analyses, as regional data cannot reflect the overall level of the country. Second, there are no available key variables (gender inequality index and Gini coefficient) for countries.
Non-participation	not applicable
Randomization	not applicable

Reporting for specific materials, systems and methods

We require information from authors about some types of materials, experimental systems and methods used in many studies. Here, indicate whether each material, system or method listed is relevant to your study. If you are not sure if a list item applies to your research, read the appropriate section before selecting a response.

Materials & experimental systems

n/a	Involved in the study
☒	☐ Antibodies
☒	☐ Eukaryotic cell lines
☒	☐ Palaeontology and archaeology
☒	☐ Animals and other organisms
☒	☐ Clinical data
☒	☐ Dual use research of concern
☒	☐ Plants

Methods

n/a	Involved in the study
☒	☐ ChIP-seq
☒	☐ Flow cytometry
☒	☐ MRI-based neuroimaging

Plants

Seed stocks	not applicable
Novel plant genotypes	not applicable
Authentication	not applicable
